# A model of inflammatory arthritis highlights a role for oncostatin M in pro-inflammatory cytokine-induced bone destruction via RANK/RANKL

**DOI:** 10.1186/ar1460

**Published:** 2004-11-10

**Authors:** Wang Hui, Tim E Cawston, Carl D Richards, Andrew D Rowan

**Affiliations:** 1 Musculoskeletal Research Group, The Medical School, University of Newcastle, Newcastle upon Tyne, UK; 2 Department of Pathology and Molecular Medicine, McMaster University, Hamilton, Ontario, Canada

**Keywords:** bone, IL-1, oncostatin M, RANK, tumour necrosis factor alpha

## Abstract

Oncostatin M is a pro-inflammatory cytokine previously shown to promote marked cartilage destruction both *in vitro *and *in vivo *when in combination with IL-1 or tumour necrosis factor alpha. However, the *in vivo *effects of these potent cytokine combinations on bone catabolism are unknown. Using adenoviral gene transfer, we have overexpressed oncostatin M in combination with either IL-1 or tumour necrosis factor alpha intra-articularly in the knees of C57BL/6 mice. Both of these combinations induced marked bone damage and markedly increased tartrate-resistant acid phosphatase-positive multinucleate cell staining in the synovium and at the front of bone erosions. Furthermore, there was increased expression of RANK and its ligand RANKL in the inflammatory cells, in inflamed synovium and in articular cartilage of knee joints treated with the cytokine combinations compared with expression in joints treated with the cytokines alone or the control. This model of inflammatory arthritis demonstrates that, *in vivo*, oncostatin M in combination with either IL-1 or tumour necrosis factor alpha represents cytokine combinations that promote bone destruction. The model also provides further evidence that increased osteoclast-like, tartrate-resistant acid phosphatase-positive staining multinucleate cells and upregulation of RANK/RANKL in joint tissues are key factors in pathological bone destruction.

## Introduction

Bone is an important skeletal extracellular matrix, and bone erosions are a major characteristic in rheumatoid arthritis (RA). The cytokines IL-1 and tumour necrosis factor (TNF) alpha play key roles in promoting joint inflammation, synovitis and cartilage/bone resorption [1,2]. These cytokines are overexpressed in RA cartilage and synovial membranes, and raised levels are found in synovial fluid and sera that correlate with disease activity and cartilage/bone destruction in RA [3-5]. Anti-IL-1 and TNF-α therapies in animal arthritis models and anti-TNF-α in humans with RA have been shown to significantly reduce arthritis incidence, inflammation and joint destruction [1,6-8], suggesting that the mediating pathways of joint damage are, at least in part, mediated by IL-1 and/or TNF-α.

Oncostatin M (OSM), a cytokine produced by activated T cells and macrophages, is structurally and functionally related to the IL-6 cytokine family. Raised levels of OSM are detected in synovial macrophages and synovial fluids of RA patients [9-11], and the levels correlate with markers of joint inflammation and destruction [3,10]. OSM causes joint inflammation, synovitis and structural damage in experimental animals [12,13]. Blockade of OSM ameliorates joint inflammation and cartilage damage in collagen-induced arthritis [14]. OSM has been found to enhance the differentiation and proliferation of osteoblasts during bone development and also induces the formation of osteoclasts and bone erosions [15-17]. These data indicate an important role for this cytokine in chronic joint inflammation and cartilage/bone damage. Furthermore, growing evidence from *in vitro *and *in vivo *studies suggests that OSM appears to be an important cofactor with other pro-inflammatory cytokines such as IL-1, TNF-α and IL-17 in mediating cartilage/bone destruction [9,18,19]. When these pro-inflammatory cytokines are overexpressed in combination with OSM in murine joints, a marked increase in damage to the joint tissues is observed [20,21].

RANKL is a TNF superfamily member and an essential mediator of osteoclastogenesis. It is produced from osteoblastic-stromal cells, synovial fibroblasts, chondrocytes and activated T lymphocytes [22,23]. This TNF-related cytokine and its receptor, RANK, are considered key factors in osteoclast differentiation, and RANK signalling is vital for osteoclast activation and survival [24,25]. RANKL binds directly to RANK on pre-osteoclasts and osteoclasts, initiating signal transduction that results in the differentiation of osteoclast progenitors as well as activation of mature osteoclasts, and therefore is implicated in the osteoclastogenic process in erosive arthritis [22,24].

The biological activity of RANKL is regulated by the soluble decoy receptor osteoprotegrin (OPG), a TNF-receptor superfamily member that is secreted by stromal cells and osteoblasts [26]. OPG competitively inhibits RANKL binding to RANK on the cell surface of osteoclast precursor cells and mature osteoclasts, thus inhibiting the osteoclastogenic actions of RANKL [27]. The levels of OPG and RANKL in osteoblastic and stromal cells are often reciprocally regulated *in vitro *and *in vivo *by bone active cytokines and hormones [28]. Excessive production of RANKL and/or deficiency of OPG may therefore contribute to the increased bone resorption typified by the focal bone erosions and peri-articular bone loss in RA.

We have recently shown in a murine model that OSM in combination with IL-1 or TNF-α synergistically promoted inflammation and cartilage degradation and increased matrix metalloproteinase expression [20,21]. Since bone erosions are also a major pathological feature of RA, we examined the effects of these cytokine combinations on bone in this model. In the present study we confirm that OSM exacerbates the effects of both IL-1 and TNF-α with respect to bone breakdown, osteoclast formation and the expression of RANK/RANKL, and further confirm that this rapid model of inflammatory arthritis is suitable for studies of RA.

## Materials and methods

### Adenoviral vectors and delivery of cytokines

Replication-defective recombinant adenoviruses engineered to overexpress murine IL-1β, TNF-α and OSM were as described previously [11,20,21], as was the empty control vector (Add170) [11]. Previous studies have validated these adenoviruses as an effective means of cytokine overexpression in synovial tissues [13,15,20,21]. All animal studies were compliant with the Canadian Council on Animal Care guidelines and were approved by the Animal Research Ethics Board at McMaster University, Canada.

C57BL/6 mice were purchased and housed until 12–14 weeks old. Mice were injected intra-articularly with adenovirus (5 × 10^6 ^plaque-forming units [pfu]/vector/joint) or PBS as previously described [13,20]. Briefly, anaesthesia was maintained with isofluorane, knees were swabbed with 70% ethanol and a 5 μl volume (treatment) was injected into the synovial space. The contralateral knee was treated with control vector or with PBS. One knee (*n *= 4 mice per treatment) was injected with combinations of vectors or with each vector alone combined with control vector to ensure that the total dose of vector was equivalent for each knee (1 × 10^7 ^pfu/joint). The animals were sacrificed at day 7 after administration.

### Histology and histopathological scoring

The whole knee joints were dissected away from the limbs and were fixed with 7% formaldehyde in phosphate buffer (pH 7.4) overnight. Subsequently, joints were decalcified in 10% EDTA in phosphate buffer (pH 7.4) for 10 days at 4°C, and were then processed for paraffin embedding and sectioning (5 μm). Sections were stained with H&E. Bone damage was rated 0–5 (0 = normal, to 5 = severely affected) according to the following semiquantitative rating scale [29]: 0, none; 1, minimal (not readily apparent on low magnification); 2, mild (more numerous areas of resorption but not readily apparent on low magnification); 3, moderate (obvious foci of resorption, numerous osteoclasts); 4, marked (large erosions extending into bone cortices, more numerous osteoclasts); and 5, extensive erosions (markedly disrupted joint architecture). All scoring was performed blind with respect to the specific treatment.

### Immunohistochemistry and tartrate-resistant acid phosphatase staining

Immunohistochemistry was performed with anti-RANK and anti-RANKL polyclonal antibodies (Santa Cruz Biotechnology, Santa Cruz, CA, USA), using the VECTASTAIN Elite ABC Kit PK 6101 (Vector, Burlingame, CA, USA). Formalin-fixed paraffin sections were deparaffinized, rehydrated and incubated with 10 mM sodium citrate, pH 6.0, for 2 hours at room temperature, and then with 3% H_2_O_2 _for 15 min. Thereafter, the sections were blocked with 1.5% normal sheep serum for 30 min, and were then incubated with primary antibody directed against RANK (rabbit polyclonal antibody raised against the epitope corresponding to amino acids 317–616 mapping at the carboxy terminus of RANK of human origin [H-300]) or against RANKL (rabbit polyclonal antibody raised against the epitope corresponding to amino acids 46–317 of RANKL of human origin [FL-317]) for 90 min at room temperature. After rinsing, sections were incubated with biotinylated secondary antibody for 30 min followed by avidin–biotin complex for 30 min according to the manufacturer's instructions (Vector). The signals were developed by 3,3'-diaminobenzidine tetrahydrochloride chromogen solution (DAKO Ltd, Ely, UK) and were counterstained with hematoxylin. A rabbit IgG antibody (X0936; Dako, Carpinteria, CA, USA) was used as a specificity control that gave no positive staining (data not shown).

Tartrate-resistant acid phosphatase (TRAP) enzyme was detected in paraffin sections (5 μm thick) using a commercial acid phosphatase leukocyte kit (Sigma, St Louis, MO, USA) according to the manufacturer's protocol.

### Statistical analysis

All data are presented as the mean ± standard error of the mean. Statistical significance was assessed by the two-tailed unpaired Student's *t *test for comparisons between the means of two groups. *P *≤ 0.05 was considered significant.

## Results

### Intra-articular overexpression of OSM in combination with either IL-1 or TNF-α induces bone damage

**Figure 1:**
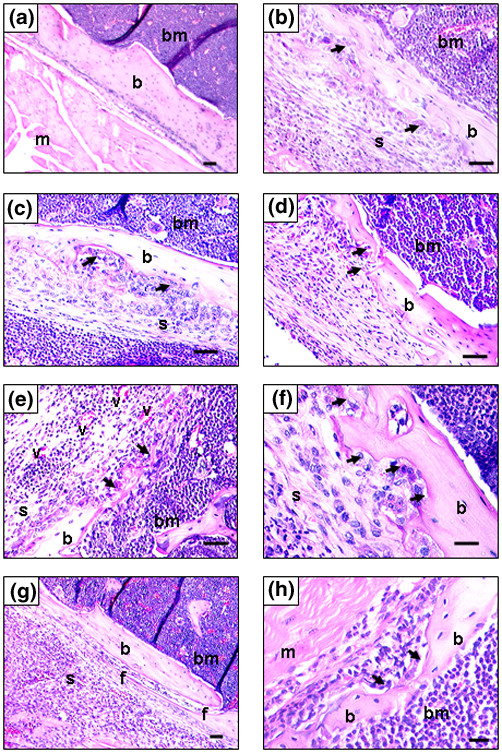
Bone damage caused by oncostatin M (OSM) in combination with either IL-1 or tumour necrosis factor alpha (TNF-α) in murine joints. Adenovirus vectors overexpressing murine OSM, IL-1 or TNF-α were injected intra-articularly into the right knee joints of mice at 5 × 10^6 ^plaque-forming units [pfu]/vector/joint. The left knee joints were injected with the empty control vector. All joints received a total of 1 × 10^7 ^pfu/joint, and all animals were sacrificed at day 7 following administration. Sections (5 μm) were stained with H&E. **(a) **Control, **(b) **OSM, **(c) **IL-1, **(d) **TNF-α, **(e), (f) **OSM + IL-1, and **(g), (h) **OSM + TNF-α. The joints showed a moderate (b–d) to severe (e–h) synovial hyperplasia and an infiltration of inflammatory cells. Marked synovial hyperplasia with angiogenesis was also seen with bone erosions (arrows) and bone fractures, with evidence of synovial invasion (e and g). b, bone; bm, bone marrow; f, bone fracture; m, muscle; s, synovial cells; v, blood vessel. (a)–(e), (g) Bar = 50 μm; (f), (h) bar = 20 μm.

The morphology of H&E-stained sections from all treated joints (5 × 10^6 ^pfu/vector/joint) was assessed, and indicated that the contralateral joints treated with the control vector showed no evidence of bone damage (Fig. 1a). Administration of each cytokine alone caused a moderate synovial hyperplasia and bone erosions (Fig. 1b,1c,1d).

For the OSM + IL-1 combination (Fig. 1e,1f), pronounced bone destruction was observed with severe synovial hyperplasia and soft tissue inflammation. Prominent features of this arthritic lesion were the marked bone erosion, especially in the area of the bone and cartilage junction where marked synovial proliferation was seen with evidence of significant angiogenesis (Fig. 1e). Some areas of bone erosion and disconnection were seen with evidence of marked synovial invasion and multinucleated cells (Fig. 1e,1f). Similar results were obtained for the OSM + TNF-α combination (Fig. 1g,1h). A number of channels developing links between the synovial tissue and the bone marrow were seen (Fig. 1g) with focal bone erosions (Fig. 1h).

**Figure 2:**
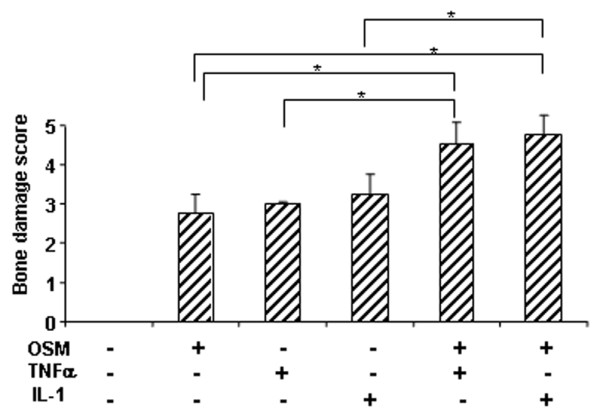
Quantitative analysis of bone damage. Mice (*n *= 4 for each treatment group) were injected with the adenoviral vectors expressing oncostatin M (OSM), IL-1 or tumour necrosis factor alpha (TNF-α) as described in Fig. 1. Sections (5 μm) were stained with H&E and were scored for bone damage as described in Materials and methods. The values represent the mean ± standard error of the mean for each treatment group (the control scored 0). The total scores are the combined scores of the four mice in each treatment group. Statistical differences between each treatment within experiments were determined using Student's *t *test: * *P *≤ 0.05.

Semiquantitative evaluations of bone damage [29] indicated an increase in the severity and extent of bone erosions with both of the cytokine combinations compared with the individual cytokines alone (*P *< 0.05) (Fig. 2).

### Intra-articular delivery of OSM with either IL-1 or TNF-α leads to an increase in TRAP-positive cells

**Figure 3:**
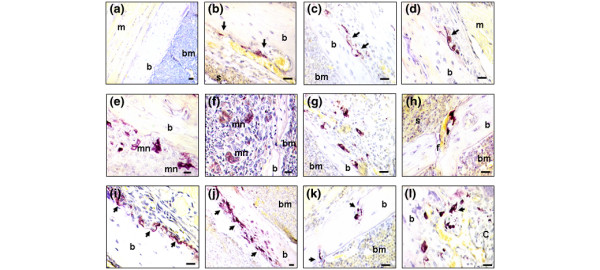
Increased tartrate-resistant acid phosphatase (TRAP)-positive staining following treatment with oncostatin M (OSM) with either IL-1 or tumour necrosis factor alpha (TNF-α) in murine joints. Mice were injected intra-articularly with adenoviral vectors as described in Fig. 1, and the animals were sacrificed at day 7 following administration. **(a) **No significant TRAP-positive cells outside the bone marrow were seen in control joints. Some TRAP-positive staining cells were located at the synovium-bone interface in **(b) **OSM-treated joints, **(c) **IL-1-treated joints and **(d) **TNF-α-treated joints. In both the **(e)**–**(h) **OSM + IL-1 and **(i)**–**(l) **OSM + TNF-α combinations, significant TRAP-positive staining was located at the front of the synovium–bone and the pannus–subchondral bone junctions, which were interposed between the bone surface and the 'erosive front' of the synovium (e, i and j). At many sites of focal bone erosions (arrows), TRAP-positive multinucleated cells were seen at the erosion front within the synovium (e, h–j), and within erosion pits in the bone (h and k). Furthermore, TRAP-positive staining cells were also seen at the cartilage/bone junction (l). b, bone; bm, bone marrow; c, cartilage; f, fracture; m, muscle; mn, multinucleated cells; s, synovial cells. (a)–(d), (g)–(l) Bar = 50 μm; (e), (f) bar = 20 μm.

No TRAP-positive staining cells were found in the control joints (Fig. 3a), but there was evidence of TRAP staining at the synovium–bone interface in joints treated with OSM, IL-1 or TNF-α (Fig. 3b,3c,3d, respectively).

When OSM was combined with IL-1 a substantial increase in the number of TRAP-positive staining cells was found at the leading edge of the synovium–bone interface (Fig. 3e), at sites within the synovium (Fig. 3f) and at the pannus–subchondral bone junction (Fig. 3g). These cells were often interposed between the bone surface and the 'erosive front' of the synovium and bone (Fig. 3h). At many sites of focal bone erosion (as in Fig. 1), TRAP-positive multinucleated cells were seen at the erosion front and in the synovium (Fig. 3e,3f), as well as in erosion pits in the bone (Fig. 3h). Furthermore, TRAP-positive staining cells were also seen in the area of cartilage/bone junctions and also in the pannus and synovium away from the eroded bone surface (Fig. 3f,3g).

Similar results were seen in the joints treated with OSM + TNF-α where TRAP-positive cells were aligned on the bone surface (Fig. 3i,3j,3k,3l). Multinucleated cells were also seen at the bone surface (Fig. 3i,3j) and in deep pockets where bone was eroded (Fig. 3k). TRAP-positive cells were also seen within the synovial tissue and the area of cartilage/bone junctions (Fig. 3l).

### Intra-articular delivery of OSM with IL-1 or TNF-α elevates RANK and RANKL expression

**Figure 4:**
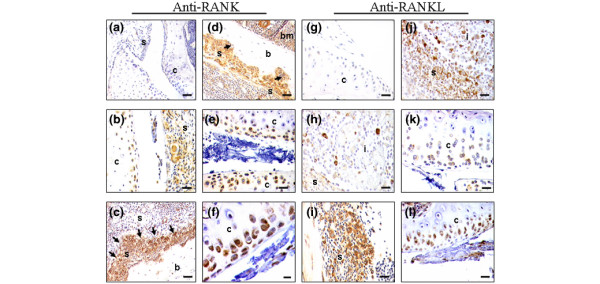
Intra-articular overexpression of oncostatin M (OSM) with either IL-1 or tumour necrosis factor alpha (TNF-α) increases RANK/RANKL expression in murine joints. Mice were injected intra-articularly with adenoviral vectors as described in Fig. 1, and the animals were sacrificed at day 7 following administration. Sections (*n *= 4 per treatment group) were immunolocalized with antibodies specific for **(a)**–**(f) **RANK or **(g)**–**(l) **RANKL with haematoxylin counterstaining. No positive staining (brown) was observed in the controls (a and g), while similar patterns of RANK and RANKL staining were observed in the synovium and inflammatory cells in joints treated with a single cytokine (representative data are shown: (b) TNF-α, (h) IL-1). This expression was further enhanced by the combinations of OSM + IL-1 (c, e, i, k) or OSM + TNF-α (d, f, j, l), especially at sites of bone erosion (arrows). A marked increase in RANK and RANKL expression was also seen in the articular chondrocytes for both cytokine combinations (e, f, k, l). b, bone; bm, bone marrow; c, cartilage; i, inflammatory cells; s, synovial cells. Bar = 50 μm.

There was little or no RANK-positive staining in synovial tissues taken from control joints (Fig. 4a). Increased RANK expression associated with inflammatory and synovial cells was observed following treatment with TNF-α (Fig. 4b), and similar levels of expression were observed for OSM and IL-1 (data not shown). RANK expression was increased further, especially at the bone erosion fronts, when OSM was combined with IL-1 or TNF-α (Fig. 4c,4d). Interestingly, RANK appeared to be expressed as a gradient from the synovial tissue where increased numbers of RANK-positive cells were observed close to the cortical bone and periosteum of the patella, the femur and the tibia. Diffuse RANK staining in the superficial layer of cartilage was seen for the joints treated with each of the vectors separately (see Fig. 4b; some data not shown), and this staining was more intense in the joints treated with OSM + IL-1 or with OSM + TNF-α (Fig. 4e,4f).

No RANKL staining was evident in the control joints, either in the cartilage (Fig. 4g) or in the synovium (data not shown). Treatment with all the individual vectors alone induced a similar positive staining for RANKL in synovial cells and in the infiltrating (inflammatory) cells (Fig. 4h). There was a marked increase in RANKL expression, consistent with the increase in inflammatory cells and synovial inflammation, in the joints treated with the combinations of OSM + IL-1 (Fig. 4i) or OSM + TNF-α (Fig. 4j). Within the articular cartilage there was very diffuse RANKL staining for both the control and each individual cytokine vector alone (data not shown), while strong RANKL expression was seen for both cytokine combinations close to the articular surface (Fig. 4k,4l).

## Discussion

In the present study, we demonstrate for the first time that overexpression of OSM in combination with either IL-1 or TNF-α causes profound bone damage with osteoclast formation and activation, and increased expression of RANK/RANKL in inflammatory cells, in inflamed synovium, in articular cartilage and at the invading front of bone erosions.

It has been long recognized that pro-inflammatory cytokines are intimately associated with bone destruction during RA. IL-1, TNF-α and OSM have all been reported to induce joint inflammation and cartilage/bone destruction *in vitro *and *in vivo *[1,2,9,13]. Elevated levels of these cytokines are found in the synovial fluid of RA patients, and these levels correlate with disease activity and cartilage/bone destruction [2,5,9,12]. Recent studies indicate that cytokines such as TNF-α and IL-1 are likely to synergize with RANKL to promote bone loss [30-33]. Indeed, TNF-α stimulates differentiation of osteoclast precursors after priming by <1% of the amount of RANKL normally required to induce osteoclast formation [31]. TNF-α induces IL-1 production, and both IL-1 and RANKL function as osteoclast survival/activation factors [33]. In the inflammatory arthritides such as RA, TNF-α and IL-1 may therefore promote bone loss by amplifying RANKL effects. This is exemplified in transgenic mice overexpressing the human TNF-α gene; these mice exhibit an erosive arthritis, which is improved by administration of OPG, of neutralizing anti-TNF-α antibodies, or of the bisphosphonate pamidronate [34]. OSM-induced expression of RANK/RANKL in a murine arthritis model has also been reported [15].

In the present study, the combination of OSM with TNF-α significantly induced RANKL expression in inflammatory cells, in inflamed synovium and in articular chondrocytes. A number of factors contribute to arthritic cartilage/bone destruction in RA, including the proliferation of synovial cells, the influx and interaction of inflammatory cells, and the maintenance of a destructive fibroblastic phenotype, which result in the final loss of cartilage and bone. Indeed, CD14^+ ^monocytes/macrophages have been shown to be osteoclast precursors within the inflamed synovium that promote bone resorption following differentiation [35]. In support of this we found evidence of high numbers of TRAP-positive multinucleate cells in the synovial tissues of mice treated with OSM + IL-1 or with OSM + TNF-α combinations.

As well as inducing marked synovial hyperplasia, angiogenesis and inflammation as described previously [20,21], marked bone erosions were also evident. Active synovial cells can cause bone erosions as well as produce factors that can themselves induce synovial proliferation, inflammation, osteoclast formation and activation. Angiogenesis contributes to synovial growth and leukocyte recruitment, thus potentiating disease progression [36]. The matrix metalloproteinases released by active synovial cells are also involved in angiogenesis, tissue invasion and inflammatory cell migration [37], as well as in osteoclast activation, migration and bone resorption [38,39]. The high levels of RANK/RANKL expression, the synovial hyperplasia, the angiogenesis and the osteoclast activity that the OSM + IL-1 or OSM + TNF-α treatments induced was associated with pronounced bone damage in this murine model with a similar pathology to that of active RA. Our previous studies have shown that these cytokine combinations upregulate matrix metalloproteinases [20,21].

TRAP is used as a molecular marker enzyme for chondroclast/osteoclast differentiation, the function of which is considered to relate to cartilage/bone resorption [40]. The cytokine combinations used in this study induced an increased number of TRAP-positive staining multinucleated cells at the pannus and cartilage/bone junctions compared with joints injected with the cytokines alone. The expression of TRAP activity by the multinucleated cells located within erosion pits and bone disconnection sites provides strong evidence of their osteoclast-like nature. The mechanism of induction of TRAP-positive multinucleated cells could be related to the marked induction of RANK/RANKL expression and the interactions between osteoblast and osteoclast precursor cells that is crucial for osteoclast development [15]. We also found that treatment with OSM increased the numbers of TRAP-positive cells at the invading front of bone erosion sites and at the bone surface, as well as in the synovium. This differs from a recent report that OSM induced synovial inflammation and increased the expression of IL-6, RANK and RANKL, but did not stimulate osteoclast activity [15]. The same authors also found marked growth plate damage, and determined that OSM-induced inflammation and proteoglycan depletion were IL-1 dependent [41].

## Conclusion

Using gene transfer technology we have provided evidence of a murine model with an aggressive pathological phenotype that closely resembles RA in terms of inflammation, angiogenesis, and cartilage and bone destruction. These data further highlight the pro-inflammatory nature of OSM and confirm a potential role for these potent cytokine combinations in the joint destruction characteristic of inflammatory arthritic diseases.

## Abbreviations

H&E = haematoxylin and eosin; IL = interleukin; OPG = osteoprotegrin; OSM = oncostatin M; PBS = phosphate buffered saline; pfu = plaque-forming units; RA = rheumatoid arthritis; RANK = receptor activator of nuclear factor kappa B; RANKL = receptor activator of nuclear factor kappa B ligand; TNF = tumour necrosis factor; TRAP, tartrate-resistant acid phosphatase.

## Competing interests

The author(s) declare that they have no competing interests.

## Authors' contributions

CDR supplied and administered the adenoviral vectors. WH performed the tissue sectioning and staining experiments. ADR drafted the manuscript and, with TEC, conceived of the study and participated in its design and coordination.

## References

[B1] Dayer JM, Bresnihan B (2002). Targeting interleukin-1 in the treatment of rheumatoid arthritis. Arthritis Rheum.

[B2] Butler DM, Malfait AM, Mason LJ, Warden PJ, Kollias G, Maini RN, Feldmann M, Brennan FM (1997). DBA/1 mice expressing the human TNF-alpha transgene develop a severe, erosive arthritis: characterization of the cytokine cascade and cellular composition. J Immunol.

[B3] Manicourt DH, Poilvache P, van Egeren A, Devogelaer JP, Lenz ME, Thonar EJ (2000). Synovial fluid levels of tumor necrosis factor alpha and oncostatin M correlate with levels of markers of the degradation of crosslinked collagen and cartilage aggrecan in rheumatoid arthritis but not in osteoarthritis. Arthritis Rheum.

[B4] Chu CQ, Field M, Allard S, Abney E, Feldmann M, Maini RN (1992). Detection of cytokines at the cartilage/pannus junction in patients with rheumatoid arthritis: implications for the role of cytokines in cartilage destruction and repair. Br J Rheumatol.

[B5] Rooney M, Symons JA, Duff GW (1990). Interleukin 1 beta in synovial fluid is related to local disease activity in rheumatoid arthritis. Rheumatol Int.

[B6] van de Loo FA, van den Berg WB (2002). Gene therapy for rheumatoid arthritis. Lessons from animal models, including studies on interleukin-4, interleukin-10, and interleukin-1 receptor antagonist as potential disease modulators. Rheum Dis Clin North Am.

[B7] Zhang HG, Xie J, Yang P, Wang Y, Xu L, Liu D, Hsu HC, Zhou T, Edwards CK, Mountz JD (2000). Adeno-associated virus production of soluble tumor necrosis factor receptor neutralizes tumor necrosis factor alpha and reduces arthritis. Hum Gene Ther.

[B8] Taylor PC, Williams RO, Maini RN (2001). Immunotherapy for rheumatoid arthritis. Curr Opin Immunol.

[B9] Cawston TE, Curry VA, Summers CA, Clark IM, Riley GP, Life PF, Spaull JR, Goldring MB, Koshy PJT, Rowan AD, Shingleton WD (1998). The role of oncostatin M in animal and human connective tissue collagen turnover and its localization within the rheumatoid joint. Arthritis Rheum.

[B10] Hui W, Bell M, Carroll G (1997). Detection of oncostatin M in synovial fluid from patients with rheumatoid arthritis. Ann Rheum Dis.

[B11] Langdon C, Leith J, Smith F, Richards CD (1997). Oncostatin M stimulates monocyte chemoattractant protein-1- and interleukin-1-induced matrix metalloproteinase-1 production by human synovial fibroblasts *in vitro*. Arthritis Rheum.

[B12] Bell MC, Carroll GJ, Chapman HM, Mills JN, Hui W (1999). Oncostatin M induces leukocyte infiltration and cartilage proteoglycan degradation *in vivo* in goat joints. Arthritis Rheum.

[B13] Langdon C, Kerr C, Hassen M, Hara T, Arsenault AL, Richards CD (2000). Murine oncostatin M stimulates mouse synovial fibroblasts *in vitro* and induces inflammation and destruction in mouse joints *in vivo*. Am J Pathol.

[B14] Plater-Zyberk C, Buckton J, Thompson S, Spaull J, Zanders E, Papworth J, Life PF (2001). Amelioration of arthritis in two murine models using antibodies to oncostatin M. Arthritis Rheum.

[B15] de Hooge AS, van de Loo FA, Bennink MB, de Jong DS, Arntz OJ, Lubberts E, Richards CD, van Den Berg WB (2002). Adenoviral transfer of murine oncostatin M elicits periosteal bone apposition in knee joints of mice, despite synovial inflammation and up-regulated expression of interleukin-6 and receptor activator of nuclear factor-kappa B ligand. Am J Pathol.

[B16] Palmqvist P, Persson E, Conaway HH, Lerner UH (2002). IL-6, leukemia inhibitory factor, and oncostatin M stimulate bone resorption and regulate the expression of receptor activator of NF-kappa B ligand, osteoprotegerin, and receptor activator of NF-kappa B in mouse calvariae. J Immunol.

[B17] Richards CD, Langdon C, Deschamps P, Pennica D, Shaughnessy SG (2000). Stimulation of osteoclast differentiation *in vitro* by mouse oncostatin M, leukaemia inhibitory factor, cardiotrophin-1 and interleukin 6: synergy with dexamethasone. Cytokine.

[B18] Hui W, Rowan AD, Cawston TE (2003). Transforming growth factor beta 1 and insulin-like growth factor 1 block collagen degradation induced by oncostatin M in combination with tumour necrosis factor alpha from bovine cartilage. Ann Rheum Dis.

[B19] Koshy PJ, Henderson N, Logan C, Life PF, Cawston TE, Rowan AD (2002). Interleukin 17 induces cartilage collagen breakdown: novel synergistic effects in combination with proinflammatory cytokines. Ann Rheum Dis.

[B20] Hui W, Richards CD, Rowan AD, Cawston TE (2003). Oncostatin M in combination with tumor necrosis factor alpha induces cartilage damage and matrix metalloproteinase expression *in vitro* and *in vivo*. Arthritis Rheum.

[B21] Rowan AD, Hui W, Cawston TE, Richards CD (2003). Adenoviral gene transfer of interleukin-1 in combination with oncostatin M induces significant joint damage in a murine model. Am J Pathol.

[B22] Jones DH, Kong YY, Penninger JM (2002). Role of RANKL and RANK in bone loss and arthritis. Ann Rheum Dis.

[B23] Takayanagi H, Iizuka H, Juji T, Nakagawa T, Yamamoto A, Miyazaki T, Koshihara Y, Oda H, Nakamura K, Tanaka S (2000). Involvement of receptor activator of nuclear factor kappaB ligand/osteoclast differentiation factor in osteoclastogenesis from synoviocytes in rheumatoid arthritis. Arthritis Rheum.

[B24] Burgess TL, Qian Y, Kaufman S, Ring BD, Van G, Capparelli C, Kelley M, Hsu H, Boyle WJ, Dunstan CR (1999). The ligand for osteoprotegerin (OPGL) directly activates mature osteoclasts. J Cell Biol.

[B25] Lacey DL, Tan HL, Lu J, Kaufman S, Van G, Qiu W, Rattan A, Scully S, Fletcher F, Juan T (2000). Osteoprotegerin ligand modulates murine osteoclast survival *in vitro* and *in vivo*. Am J Pathol.

[B26] Simonet WS, Lacey DL, Dunstan CR, Kelley M, Chang MS, Luthy R, Nguyen HQ, Wooden S, Bennett L, Boone T (1997). Osteoprotegerin: a novel secreted protein involved in the regulation of bone density. Cell.

[B27] Hsu H, Lacey DL, Dunstan CR, Solovyev I, Colombero A, Timms E, Tan HL, Elliott G, Kelley MJ, Sarosi I (1999). Tumor necrosis factor receptor family member RANK mediates osteoclast differentiation and activation induced by osteoprotegerin ligand. Proc Natl Acad Sci USA.

[B28] Kobayashi K, Takahashi N, Jimi E, Udagawa N, Takami M, Kotake S, Nakagawa N, Kinosaki M, Yamaguchi K, Shima N (2000). Tumor necrosis factor alpha stimulates osteoclast differentiation by a mechanism independent of the ODF/RANKL–RANK interaction. J Exp Med.

[B29] Romas E, Sims NA, Hards DK, Lindsay M, Quinn JW, Ryan PF, Dunstan CR, Martin TJ, Gillespie MT (2002). Osteoprotegerin reduces osteoclast numbers and prevents bone erosion in collagen-induced arthritis. Am J Pathol.

[B30] Kotake S, Udagawa N, Takahashi N, Matsuzaki K, Itoh K, Ishiyama S, Saito S, Inoue K, Kamatani N, Gillespie MT (1999). IL-17 in synovial fluids from patients with rheumatoid arthritis is a potent stimulator of osteoclastogenesis. J Clin Invest.

[B31] Lam J, Takeshita S, Barker JE, Kanagawa O, Ross FP, Teitelbaum SL (2000). TNFα induces osteoclastogenesis by direct stimulation of macrophages exposed to permissive levels of RANK ligand. J Clin Invest.

[B32] Zhang YH, Heulsmann A, Tondravi MM, Mukherjee A, Abu-Amer Y (2001). Tumor necrosis factor-α (TNF) stimulates RANKL-induced osteoclastogenesis via coupling of TNF type 1 receptor and RANK signaling pathways. J Biol Chem.

[B33] Feige U, Hu YL, Gasser J, Campagnuolo G, Munyakazi L, Bolon B (2000). Anti-interleukin-1 and anti-tumor necrosis factor-α synergistically inhibit adjuvant arthritis in Lewis rats. Cell Mol Life Sci.

[B34] Schett G, Redlich K, Hayer S, Zwerina J, Bolon B, Dunstan C, Gortz B, Schulz A, Bergmeister H, Kollias G (2003). Osteoprotegerin protects against generalized bone loss in tumor necrosis factor-transgenic mice. Arthritis Rheum.

[B35] Danks L, Sabokbar A, Gundle R, Athanasou NA (2002). Synovial macrophage-osteoclast differentiation in inflammatory arthritis. Ann Rheum Dis.

[B36] Stupack DG, Storgard CM, Cheresh DA (1999). A role for angiogenesis in rheumatoid arthritis. Braz J Med Biol Res.

[B37] Chang C, Werb Z (2001). The many faces of metalloproteases: cell growth, invasion, angiogenesis and metastasis. Trends Cell Biol.

[B38] Goto T, Maeda H, Tanaka T (2002). A selective inhibitor of matrix metalloproteinases inhibits the migration of isolated osteoclasts by increasing the life span of podosomes. J Bone Miner Metab.

[B39] Hill PA, Murphy G, Docherty AJ, Hembry RM, Millican TA, Reynolds JJ, Meikle MC (1994). The effects of selective inhibitors of matrix metalloproteinases (MMPs) on bone resorption and the identification of MMPs and TIMP-1 in isolated osteoclasts. J Cell Sci.

[B40] Leisen JC, Duncan H, Riddle JM, Pitchford WC (1988). The erosive front: a topographic study of the junction between the pannus and the subchondral plate in the macerated rheumatoid metacarpal head. J Rheumatol.

[B41] de Hooge AS, van de Loo FA, Bennink MB, Arntz OJ, Fiselier TJ, Franssen MJ, Joosten LA, Van Lent PL, Richards CD, van den Berg WB (2003). Growth plate damage, a feature of juvenile idiopathic arthritis, can be induced by adenoviral gene transfer of oncostatin M: a comparative study in gene-deficient mice. Arthritis Rheum.

